# The electronic and optical properties of quaternary GaAs_1-*x*-*y*_N_*x*_Bi_*y*_ alloy lattice-matched to GaAs: a first-principles study

**DOI:** 10.1186/1556-276X-9-580

**Published:** 2014-10-18

**Authors:** Xiaoyang Ma, Dechun Li, Shengzhi Zhao, Guiqiu Li, Kejian Yang

**Affiliations:** 1 School of Information Science and Engineering, Shandong University, Jinan 250100, China

**Keywords:** GaAs_1-*x*-*y*_N_*x*_Bi_*y*_, First-principles, Hybrid functional, Electronic structures, Optical properties

## Abstract

First-principles calculations based on density functional theory have been performed for the quaternary GaAs_1-*x*-*y*_N_*x*_Bi_*y*_ alloy lattice-matched to GaAs. Using the state-of-the-art computational method with the Heyd-Scuseria-Ernzerhof (HSE) hybrid functional, electronic, and optical properties were obtained, including band structures, density of states (DOSs), dielectric function, absorption coefficient, refractive index, energy loss function, and reflectivity. It is found that the lattice constant of GaAs_1-*x*-*y*_N_*x*_Bi_*y*_ alloy with *y*/*x* =1.718 can match to GaAs. With the incorporation of N and Bi into GaAs, the band gap of GaAs_1-*x*-*y*_N_*x*_Bi_*y*_ becomes small and remains direct. The calculated optical properties indicate that GaAs_1-*x*-*y*_N_*x*_Bi_*y*_ has higher optical efficiency as it has less energy loss than GaAs. In addition, it is also found that the electronic and optical properties of GaAs_1-*x*-*y*_N_*x*_Bi_*y*_ alloy can be further controlled by tuning the N and Bi compositions in this alloy. These results suggest promising applications of GaAs_1-*x*-*y*_N_*x*_Bi_*y*_ quaternary alloys in optoelectronic devices.

## Background

In recent years, the synthesis of semiconductor alloys with specific structures, particularly electronic, and optical properties is widely demanded in the application of photoelectric devices, and a great deal of effort has been devoted to explore some nonconventional alloys, especially for III-V compound semiconductors. For example, InGaAsP, BInGaAs, and BGaAsSb have been investigated adequately from the aspects of structure, electronic, and optical properties for their potential applications in lasers, detectors, solar cells, etc. [1-5]. A GaAs alloy containing a few percents of N has significant effects on both lowering the lattice constant and narrowing the band gap. However, due to the large atomic size mismatch between As and N, it is difficult to grow high-quality GaAs_1-*x*_N_*x*_ alloy on GaAs substrates. In order to overcome the problem, the coalloying approach is proposed. By substituting large-atom X, the new alloy XGaAsN can be made lattice-matched to GaAs. Most of the previous research works put emphasis on In_*y*_Ga_1-*y*_As_1-*x*_N_*x*_ grown on a GaAs substrate. However, the alloy quality deteriorates very fast when the N concentration increases, suggesting as very low photoluminescence efficiency and very short diffusion length. These impede the use of In_*x*_Ga_1-*x*_As_1-*y*_N_*y*_ in device applications [6,7]. Considering that coalloying Bi with N in GaAs can significantly lower the N concentration, which is required to reduce the band gap of alloy XGaAsN, and the strain compensation between the small-sized N and the large-sized Bi atoms also can reduce formation energies of the alloy [6], GaAs_1-*x*-*y*_N_*x*_Bi_*y*_ is proposed as a new potential III-V compound semiconductors in optoelectronic devices such as 1.06-μm solid-state lasers, high-efficiency multijunction solar cells, and so on. Therefore, it is essential to have more comprehensive investigations on the fundamental physical properties of GaAsNBi, such as structural, electronic, and optical properties (dielectric function, absorption coefficient, refractive index, energy loss function, and reflectivity).

It is well known that density functional theory (DFT) using the local density approximation (LDA) or the generalized gradient approximation (GGA) often severely underestimates the band gap, so it is much better to apply a hybrid functional to correct the band gap underestimation in the first-principles calculations [8-10]. In this paper, by first-principles calculations based on DFT as implemented in the Vienna *ab initio* simulation package (VASP) code with a modified Heyd-Scuseria-Ernzerhof (HSE) hybrid exchange-correlation functional, band structures and density of states were calculated. Besides, we also have calculated the optical parameters (absorption, refractive index, energy loss function, and reflectivity) of GaAs and GaAs_1-*x*-*y*_N_*x*_Bi_*y*_ quaternary alloys. In addition, the influence of doping concentration on electronic and optical properties of the compound semiconductor has also been obtained.

## Methods

All calculations were performed by using VASP with projector-augmented wave (PAW) for the interaction between electrons and ions and Perdew-Burke-Ernzerhof (PBE)-based HSE functional for the exchange-correlation functionals. The cutoff energy for the plane-wave expansion was set to 400 eV. The first Brillouin zone was sampled by a Г-centered 2 × 2 × 2 mesh for the supercells. A small Gaussian broadening *σ* =0.05 eV was applied so that the peaks of the defect cannot merge with the band continuum for the calculations of density of states (DOS). In the calculations of band structure and optical properties, k-point meshes were replaced by high symmetry point which was set manually according to the Brillouin zone path. All the results were obtained on the basis of the convergences. The supercell of GaAs with 64 atoms was adopted in all calculations, in which partial As atoms were replaced by the doped atoms of N or Bi. It has been validated that the dopant positions have insignificant influence on the lattice parameters by our calculated results. A crystal structure of cubic face-centered type with the space group symmetry F-43 M was adopted. And the whole system was electronic neutrality. In addition, all structures were fully optimized until the force on each atom is smaller than 0.01 eV/Å.

## Results and discussion

### Structures and electronic properties

By the well-known Vegard's law, lattice parameter of GaAs_1-*x*-*y*_N_*x*_Bi_*y*_ alloy can be expressed as

(1) $$a\left(x,y\right)=\left(1-x-y\right)a_{\text{GaAs}}+xa_{\text{GaN}}+ya_{\text{GaBi}}$$

Our calculated lattice parameters of GaAs, GaN, and GaBi are 5.653, 4.586, and 6.478, respectively, which are in good agreement with experimental values of 5.653, 4.50, and 6.234 [6]. According to our calculated results, the requirement for GaAs_1-*x*-*y*_N_*x*_Bi_*y*_ lattice-matched to GaAs shows that ratio between the Bi and N composition *y*/*x* is 1.718, in good agreement with the published literature [6]. As the method of virtual crystal approximation has some queries, the supercell approach with 64 atoms was adopted, in which partial As atoms were replaced by the doped atoms N or Bi atoms. However, it is too hard to reach the accurate ratio 1.718. From our calculated results, the lattice parameter of GaAs_1-*x*-*y*_N_*x*_Bi_*y*_ is about 11.297 when *x* =1/32 and *y* =1/16, very close to the lattice parameter of GaAs supercell 11.306. Therefore, we assume that the quaternary alloy GaAs_1-*x*-*y*_N_*x*_Bi_*y*_ (*x* =1/32 and *y* =1/16) can be made lattice-matched to GaAs approximately, and we will discuss its properties in details.

**Figure 1:**
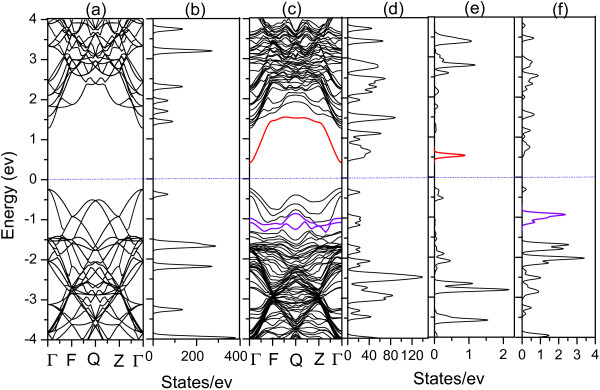
**Band structure and total DOS. (a, b)**The band structure and total DOS of perfect GaAs. **(c, d)** The band structure and total DOS of GaAs_1-*x*-*y*_N_*x*_Bi_*y*_ (*x* =1/32, *y* =1/16). **(e)** The L-DOS of N. **(f)** L-DOS of Bi.

The band structures and DOS of perfect GaAs and quaternary alloy GaAs_1-*x*-*y*_N_*x*_Bi_*y*_ (*x* =1/32 and *y* =1/16) are presented in Figure 1. Obviously, the conduction band minimum and valence band maximum in Figure 1c are both located at Г point, which means that GaAs_1-*x*-*y*_N_*x*_Bi_*y*_ alloys have the direct band gap at this composition. Figure 1a shows the band structure of perfect GaAs, our calculated band gap of GaAs is 1.51 eV, in good agreement with its experimental value. Compared with GaAs, the band gap of GaAs_1-*x*-*y*_N_*x*_Bi_*y*_ in Figure 1c reduced significantly. It can be obtained that the band gap of GaAs_1-*x*-*y*_N_*x*_Bi_y_ is 0.725 eV, which is corresponding to the wavelength of 1.7 μm. Therefore, the lattice-matched GaAs_1-*x*-*y*_N_*x*_Bi_*y*_ alloy can be applied to wavelength shorter than 1.7 μm in optoelectronic devices. As can be seen from the projected DOS of GaAs_1-*x*-*y*_N_*x*_Bi_*y*_ in Figure 1e,f, N doping mainly contributes to the conduction band, leading to the conduction band minimum moves towards the Fermi energy (the red line, corresponding to the red peak), and Bi doping mainly contributes to the valence band, resulting in a reduced band gap due to the intraband level repulsions (the violet lines, corresponding to the violet peaks), consistent with the previous research [10]. And the interaction between them contributes to the band gap reduction of GaAs_1-*x*-*y*_N_*x*_Bi_*y*_.

### Optical properties

Optical properties of GaAs_1-*x*-*y*_N_*x*_Bi_*y*_ (*x* =1/32 and *y* =1/16) alloy lattice-matched to GaAs have also been studied. The characteristics of optical properties can be described in terms of the dielectric function *ε*(*ω*) = *ε*_1_(*ω*) + *iε*_2_(*ω*). *ε*(*ω*) is an important parameter for semiconductors, which can be used to describe the interaction between electrons and photons. The imaginary part can be obtained from the momentum matrix elements between the unoccupied and occupied wave functions within the selection rules, and the real part can be calculated from the imaginary part by Kramers-Kronig correlations. The optical constants, such as absorption coefficient *α*(*ω*), refractive index *n*(*ω*), energy loss function *L*(*ω*), and reflectivity *R*(*ω*) are very important for the optical materials and related applications. These constants can be determined with the components of dielectric tensor:

(2) $$\alpha \left(\omega \right)=\sqrt{2}\omega \left[\sqrt{\varepsilon_{1}^{2}\left(\omega \right)+\varepsilon_{2}^{2}\left(\omega \right)}-\varepsilon_{1}\left(\omega \right)\right]$$

(3) $$n\left(\omega \right)=\frac{1}{\sqrt{2}}{\left[\sqrt{\varepsilon_{1}^{2}\left(\omega \right)+\varepsilon_{2}^{2}\left(\omega \right)}+\varepsilon_{1}\left(\omega \right)\right]}^{1/2}$$

(4) $$L\left(\omega \right)=\frac{\varepsilon_{2}\left(\omega \right)}{\left[\varepsilon_{1}^{2}\left(\omega \right)+\varepsilon_{2}^{2}\left(\omega \right)\right]}$$

(5) $$R\left(\omega \right)={\left|\frac{\sqrt{\varepsilon_{1}\left(\omega \right)+i\varepsilon_{2}\left(\omega \right)}-1}{\sqrt{\varepsilon_{1}\left(\omega \right)+i\varepsilon_{2}\left(\omega \right)}+1}\right|}^{2}$$

**Figure 2:**
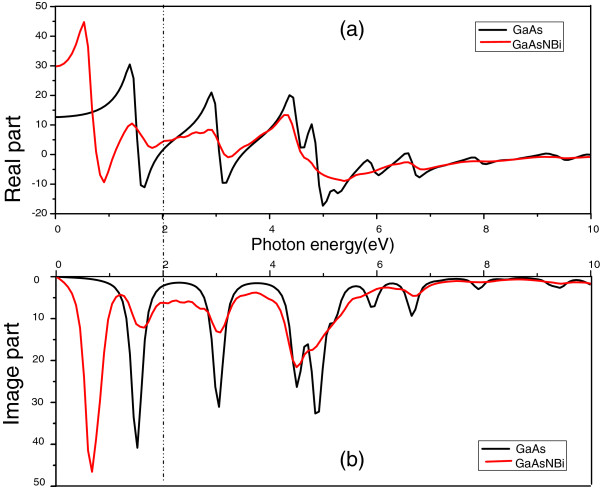
The real (a) and imaginary parts (b) of dielectric function where the black lines represent GaAs and the red lines represent GaAsNBi.

In Figure 2, the real and imaginary components of dielectric function are given as a function of the photon energy for GaAs (black curves) and quaternary alloy GaAsN_1/32_Bi_1/16_ (red curves). For GaAs, the calculated static dielectric constant *ε*_1_(0) is 12.68, very close to experimental value of 12.9 [11]. And for GaAsN_1/32_Bi_1/16_, the calculated static dielectric constant is 29.81, much larger than GaAs, due to adding N and Bi atoms to the GaAs structure.

As can be seen from Figure 2, the real part of the dielectric function of GaAs is not a flat curve. It increases with the increase of photon energy in some regions, which means normal dispersion properties. Whereas in the other regions, it decreases as the photon energy increases and this is an abnormal dispersion characteristic. The same is true for the real part of GaAsN_1/32_Bi_1/16_. Considering the actual application situation, the properties of low photon energies 0 to 2 eV will be discussed in detail.

In the low photon energies 0 to 2 eV, for GaAs, the real part of dielectric function shows an increase with the increase of photon energy in 0 to 1.38 eV and 1.65 to 2 eV, acting as normal dispersion. While it decreases, the photon energy increases from 1.38 to 1.65 eV, acting as abnormal dispersion. Besides, the imaginary part of dielectric function has one major peak at 1.526 eV in the low photon energies 0 to 2 eV. For GaAsN_1/32_Bi_1/16_, the real component shows an increase with the increase of photon energy in 0 to 0.52, 0.89 to 1.4, and 1.79 to 2 eV, whereas it decreases as the photon energy increases in 0.52 to 0.89 and 1.4 to 1.79 eV. In addition, the imaginary part of dielectric function has two major peaks in the low photon energies 0 to 2 eV, consistent to the decreases of the real part. Furthermore, the red curves obviously shift towards the lower photon energy in Figure 2, meaning that both the real and imaginary parts of GaAs_29/32_ N_1/32_Bi_1/16_ have redshifted.

It can be observed that for GaAsN_1/32_Bi_1/16_, when the photon energy is close to 1 eV, the real and image part are both near the turning point in the rise and fall, which means that the wavelength corresponding to the energy about 1 eV is far away from the resonance absorption area, avoiding great resonance absorption at this wavelength.

**Figure 3:**
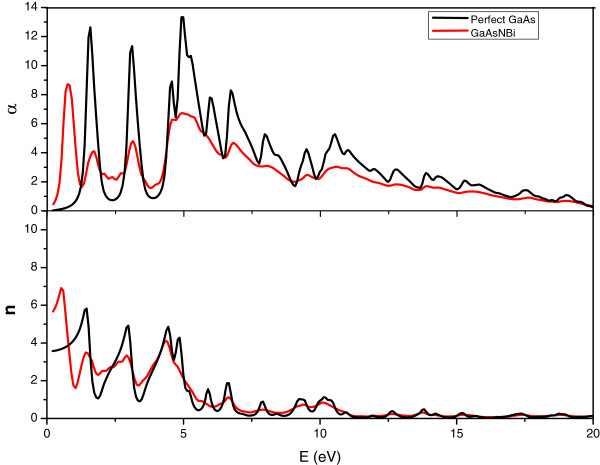
**Absorption coefficient and refractive index.** The absorption coefficient *α*(*ω*) and refractive index *n*(*ω*) of GaAs (black lines) and GaAs_1-*x*-*y*_N_*x*_Bi_*y*_ (red lines) with *x* =1/32, *y* =1/16.

According to the above formulas (2) to (5), the absorption coefficient *α*(*ω*), refractive index *n*(*ω*), energy loss function *L*(*ω*), and reflectivity *R*(*ω*) can be obtained. As can be seen from Figure 3, at the absorption onset, the absorption of GaAs is similar to that of GaAs_1-*x*-*y*_N_*x*_Bi_*y*_; however, the GaAs absorption is much stronger when the photon energy increases. Their tendencies are in correspondence to the imaginary parts. In addition, the first absorption peak of GaAs_1-*x*-*y*_N_*x*_Bi_*y*_ is at 0.73 eV, very close to the band gap 0.72 eV. Similarly, the first absorption peak of GaAs is at 1.49 eV, close to the band gap 1.51 eV. The electronic transition between the valance band and conduction band is more likely to occur in GaAs_1-*x*-*y*_N_*x*_Bi_*y*_, and it is known that the transition will be reflected in the optical properties, so the doping of Bi and N caused the change of the optical properties.

The refractive index shows an appreciable value in low-energy region and a considerable reduction in high-energy region. Besides, the static refractive index value of GaAs is 3.56, which is in good agreement with the experimental data 3.30 [12]. The static refractive index value of GaAs_1-*x*-*y*_N_*x*_Bi_*y*_ which is 5.46, lager than the value of GaAs, represents more rays that can be accessed into GaAs_1-*x*-*y*_N_*x*_Bi_*y*_ than GaAs.

**Figure 4:**
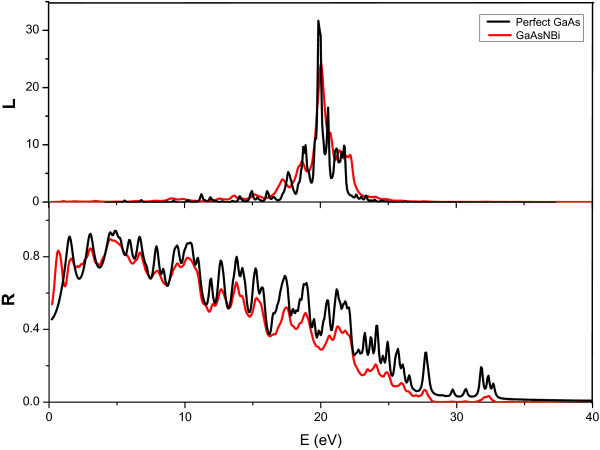
**The energy loss function *L*** **(*ω*) and reflectivity *R*** **(*ω*) of GaAs (black lines) and GaAs**_**1-** ***x*** **-** ***y***_**N**_***x***_**Bi**_***y ***_**(red lines) with *x *** **=1/32, *y *** **=1/16.**

For GaAs_1-*x*-*y*_N_*x*_Bi_*y*_, with the incorporation of N and Bi, the absorption coefficient *α*(*ω*), refractive index *n*(*ω*), and reflectivity *R*(*ω*) are all obviously redshifted. The values of energy loss function and reflectivity also have been obtained, as shown in Figure 4. Energy loss function defines the energy loss of the electrons passing between bands. The peak of energy loss function is related to plasma frequency, above which the material behaves like dielectric while below which the material exhibits the metallic property [13]. Compare to GaAs, the peak value of energy loss function of GaAs_1-*x*-*y*_N_*x*_Bi_*y*_ is obviously smaller, which means that GaAs_1-*x*-*y*_N_*x*_Bi_*y*_ has much less energy loss. So it can be concluded that energy be used more effectively in GaAs_1-*x*-*y*_N_*x*_Bi_*y*_. In addition, the peak of the energy loss spectra also corresponds to the trailing edges in the reflection spectra [14]. The value of reflectivity of GaAs is appreciable in low-energy region and decreases with the increment of the photon energy, which is consistent with the change of the value of GaAs_1-*x*-*y*_N_*x*_Bi_*y*_.

### The properties of GaAs_1-*x*-*y*_N_*x*_Bi_*y*_ with different Bi (or N) composition

In order to better understand the influence of N and Bi composition, the properties of GaAs_1-*x*-*y*_N_*x*_Bi_*y*_ with different Bi compositions or different N compositions have been calculated respectively. According to the Hume-Rothery size rule, the total concentration of N and Bi which we calculated is limited within 25%.

**Figure 5:**
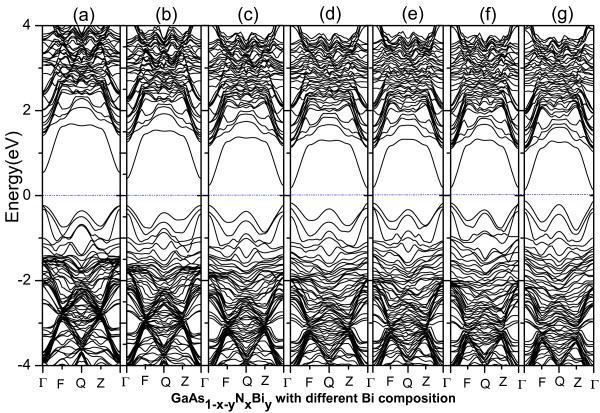
**The band structures of GaAs**_**1-*x*-*y***_**N**_***x***_**Bi**_***y***_ **with different Bi compositions.** *x* =1/32; **(a)**. *y* =1/32; **(b)**. *y* =1/16; **(c)**.* y* =3/32; **(d)**. *y* =1/8; **(e)**. *y* =5/32; **(f)**. *y* =3/16; **(g)**. *y* =7/32.

**Figure 6:**
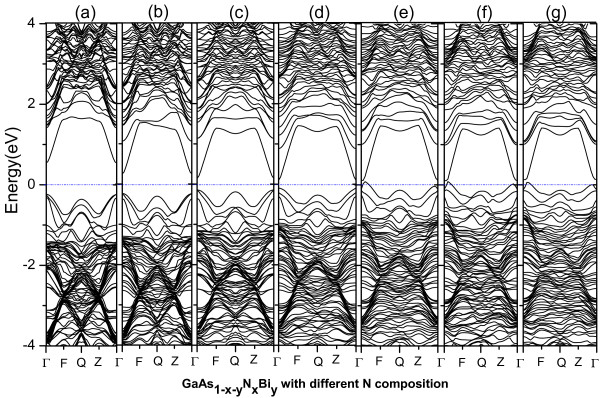
**The band structures of GaAs**_**1- *x *-*y***_**N**_***x***_**Bi**_***y ***_**with different N compositions.** *y* =1/32; **(a)**. *x* =1/32; **(b)**. *x* =1/16; **(c)**. *x* =3/32; **(d)**. *x* =1/81; **(e)**. *x* =5/32; **(f)**. *x* =3/16; **(g)**. *x* =7/32.

As can be seen from Figure 5, with the increase of the concentration of Bi, the band gap of GaAs_1-*x*-*y*_N_*x*_Bi_*y*_ has a significant reduction. And when the concentration reaches to 5/32, the band gap reduction becomes saturated. The same as Figure 5, with the increase of the concentration of N, the band gap of GaAs_1-*x*-*y*_N_*x*_Bi_*y*_ also decreases greatly in Figure 6, and Figure 6e,f,g shows that when the concentration of N increases to 5/32, the band gap of GaAs_1-*x*-*y*_N_*x*_Bi_*y*_ also becomes zero, and the quaternary alloys exhibit metallic alloy. In addition, DOSs changes of different doping configurations are consistent with those of the band structures.

**Figure 7:**
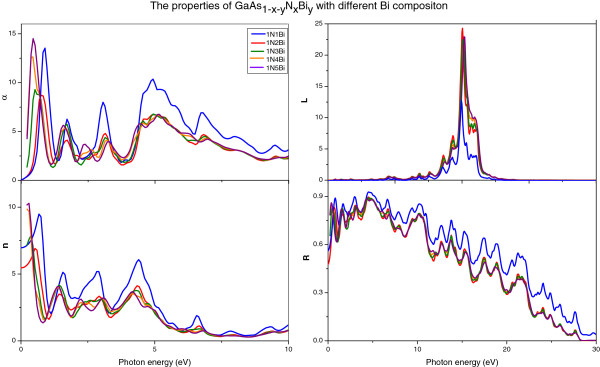
***α*** **(*ω*), *n*** **(*ω*), *L*** **(*ω*), *R*** **(*ω*) of GaAs**_**1-** ***x*** **-** ***y***_**N**_***x***_**Bi**_***y ***_**with different Bi compositions.**

The absorption coefficient *α*(*ω*), refractive index *n*(*ω*), energy loss function *L*(*ω*), and reflectivity *R*(*ω*) of GaAs_1-*x*-*y*_N_*x*_Bi_*y*_ with different Bi compositions are shown in Figure 7; with the increase of Bi composition, they all have redshift except energy loss function *L*(*ω*). The value of absorption coefficient *α*(*ω*), refractive index *n*(*ω*), and reflectivity *R*(*ω*) for GaAs_1-*x*-*y*_N_*x*_Bi_*y*_ with different Bi compositions are appreciable in low-energy region. In addition, it can be seen that when the Bi composition reaches 1/16, the optical properties of GaAs_1-*x*-*y*_N_*x*_Bi_*y*_ have minor changes with the augment of Bi composition, which means that Bi composition have less effects on the optical properties after reach a certain degree.

**Figure 8:**
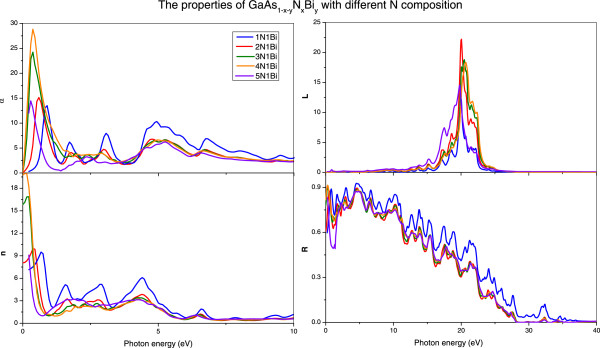
***α*** **(*ω*), *n*** **(*ω*), *L*** **(*ω*), *R*** **(*ω*) of GaAs**_**1-** ***x*** **-** ***y***_**N**_***x***_**Bi**_***y ***_**with different N compositions.**

Figure 8 shows the absorption coefficient *α*(*ω*), refractive index *n*(*ω*), energy loss function *L*(*ω*), and reflectivity *R*(*ω*) of GaAs_1-*x*-*y*_N_*x*_Bi_*y*_ with different N compositions. The same as Figure 6, with the increase of N composition, they also have redshift except energy loss function *L*(*ω*). The value of absorption coefficient *α*(*ω*), refractive index *n*(*ω*), and reflectivity *R*(*ω*) for GaAs_1-*x*-*y*_N_*x*_Bi_*y*_ with different N compositions are appreciable in low-energy region. Furthermore, it can be seen that, when the N composition between 1/16 and 1/8, the optical properties of GaAs_1-*x*-*y*_N_*x*_Bi_*y*_ have minor changes with the augment of N composition, which means that N composition has less of an effect on the optical properties in this range. When the N composition reaches 5/32, the curves of *L*(*ω*) and *R*(*ω*) have some abnormal changes; this may be caused by the metallic properties of GaAs_1-*x*-*y*_N_*x*_Bi_*y*_ quaternary alloys when the concentration of N achieves to 5/32. In general, both Bi and N have significant impact on the electronic and optical properties.

## Conclusions

In summary, the effects of N and Bi incorporation on the electronic and optical properties of GaAs have been systematically studied based on the first-principles calculations. GaAs_1-*x*-*y*_N_*x*_Bi_*y*_ alloy with the ratio *y*/*x* =1.718 lattice-matched to GaAs.

(i) With the incorporation of N and Bi into GaAs, the band gap of GaAs_1-*x*-*y*_N_*x*_Bi_*y*_ lattice-matched to GaAs has a significant reduction. It is found that N doping mainly contributes to the conduction band and Bi doping mainly contributes to the valence band.

(ii) The optical properties of GaAs_1-*x*-*y*_N_*x*_Bi_*y*_ lattice-matched to GaAs have also been calculated. It can be seen from the dielectric function that when the photon energy is close to 1 eV, the real and image part are both near the turning point in the rise and fall. That means the wavelength corresponding to 1 eV is far away from the resonance absorption area. In addition, from the calculated results of optical properties, more rays can be accessed into GaAs_1-*x*-*y*_N_*x*_Bi_*y*_ than GaAs and GaAs_1-*x*-*y*_N_*x*_Bi_*y*_ has less energy loss.

(iii) The effects of different Bi and N compositions on the electronic and optical properties have also been systematically studied, including the band structure, absorption coefficient, reflectivity, refractive index, and energy loss function. The calculated results indicate the significant change induced by the incorporation of Bi and N.

It is believed that our calculated results will be useful for the device applications of GaAs_1-*x*-*y*_N_*x*_Bi_*y*_ quaternary alloys especially in optoelectronic devices such as solid-state lasers, high-efficiency multijunction solar cells, and so on.

## Competing interests

The authors declare that they have no competing interests.

## Authors’ contributions

XYM carried out the calculations and wrote the most part of the article. DCL supervised the calculations and contributed to the writing of the article. SZZ, GQL, and KJY have polished the articles. All authors read and approved the final manuscript.
